# Theabrownin from Wuniuzao Dark Tea Regulates Hepatic Lipid Metabolism and Gut Microbiota in Mice Fed High-Fat Diet

**DOI:** 10.3390/nu15234912

**Published:** 2023-11-24

**Authors:** Qianqian Xu, Jiangcheng Ye, Mingxiu Gong, Yifan Zhang, Yiwei Yuan, Jin Zhao

**Affiliations:** Key Laboratory of Specialty Agri-Product Quality and Hazard Controlling Technology of Zhejiang Province, Institute of Food Nutrition and Quality Safety, College of Life Sciences, China Jiliang University, Hangzhou 310018, China; qianqianxu@zju.edu.cn (Q.X.); s20090710065@cjlu.edu.cn (J.Y.); p21091055012@cjlu.edu.cn (M.G.); s21090710071@cjlu.edu.cn (Y.Z.); p22091055068@cjlu.edu.cn (Y.Y.)

**Keywords:** theabrownin, lipid metabolism, gut microbiota, high-fat diet

## Abstract

The search for functional foods with no side effects that can alleviate obesity has been a common trend. Wuniuzao dark tea could be a safe choice. This study aimed to explore whether theabrownin from Wuniuzao dark tea could regulate hepatic lipid metabolism and gut microbiota in mice fed a high-fat diet. In total, fifty 8-week-old male C57BL/6 mice were randomly divided into five treatment groups, including a normal control group, high-fat diet group, positive control group, low-dose theabrownin group, and high-dose theabrownin group. After a 9-week intervention, these mice were selected from each treatment group for sampling. The results showed that the body weight and epididymis fat weight of obese mice fed with theabrownin were decreased. Serum total triglycerides, total cholesterol, and low-density lipoprotein cholesterol, and activities of aspartate aminotransferase and alanine aminotransferase were also decreased. Protein and mRNA expression of fatty acid synthesis and lipid production-related genes of mice fed with theabrownin were downregulated. The gut microbiota composition in the theabrownin group was improved. The study indicated that theabrownin from Wuniuzao dark tea could achieve the liver protection and anti-obesity effects by regulating the *Srebp* lipid metabolism pathway and bile acid metabolism process, and improving the gut microbiota composition of mice.

## 1. Introduction

The obesity as a multifactorial metabolic condition which induce hypercholesterolemia, hyperlipidemia, and steatohepatitis has become a major menace to global health [1]. Previously, pharmacological interventions are frequently employed in clinical settings and have demonstrated short-term efficacy in weight reduction and lipid level modulation. Nevertheless, as we all known, long-term use of the medications could elicit serious adverse effects on human body [2]. Dietary modifications and physical exercise are regarded as the most efficacious means for preventing and managing obesity [3]. Given the time and effort required to exercise, dietary regulation seems to be easier. The search for a functional food with no side effects that can alleviate obesity has been a general trend.

Tea, as a natural plant beverage, is considered a safe choice. Depending on the processing technique and fermentation level, tea can be categorized into six distinct types including green tea, white tea, yellow tea, oolong tea, black tea, and dark tea [4]. Each type of tea has its own unique characteristics in terms of color, aroma and flavor due to its different components [5]. Variant major biological active substances in different tea result in slightly different functional effects of tea. Comparative studies in rats treated with several different types of tea provided supporting evidence that fully fermented dark tea is more effective in the attenuation or reversal of hypercholesterolemia, hyperlipidemia, steatohepatitis, and obesity compared to other partially fermented and non-fermented tea [6]. Recently, dark tea distinguished by its unique microbial solid-state fermentation process has gained recognition as a novel approach for treating and intervening in obesity [7]. The current research on dark tea mainly focuses on the ripe Pu-erh tea, Liupao tea, Sichuan dark tea and Fuzhuan brick tea. These dark teas have garnered increasing attention for their diverse health benefits, including their potential in preventing hypertension and cardiovascular diseases [8], managing diabetes [9], and regulating the gut microbiota [10]. However, research on the biological functions of Wuniuzao dark tea and its components is rather limited, especially in terms of its effect on alleviating obesity. Therefore, we put forward the questions of whether Wuniuzao dark tea could also play a role in alleviating obesity, and what its specific mechanism is.

With the deepening of the research on the health care function of tea components, tea pigment has received extensive attention and become a hot topic in the world. During the fermentation process of tea leaves, catechins and their gallate derivatives are oxidized to complex phenolic tea pigments which include theabrownin, thearubigins, and theaflavin. Theaflavins undergo further oxidation to form more polymerized theaflavins, which are then concentrated into theabrownin [11]. It has been observed that dark tea exhibits a remarkable abundance of theabrownin. Previous studies on ripe Pu-er tea have indicated that theabrownin is the characteristic component of dark tea, which may be the bioactive substance that help dark tea carry out hypocholesterolemic and hypolipidemic functions, and may be used in order to fix the obesity epidemic [12].

Therefore, given that a high-fat diet can induce obesity, we hypothesize that theabrownin derived from Wuniuzao dark tea could alleviate obesity by regulating lipid metabolism and gut microbiota in mice fed a high-fat diet. To verify this hypothesis, we determined the effects of theabrownin on body weight, serum lipid levels, liver damage indicators, hepatic lipid deposition, mRNA and protein expression of lipid metabolism related key genes, and gut microbiota alterations in high-fat diet-induced obese mice.

## 2. Materials and Methods

All experimental protocols involving animals were approved by the Animal Care and Welfare Committee of Life Science College and the Scientific Ethical Committee of China Jiliang University (No. CJLU2023001; Hangzhou, China).

### 2.1. Theabrownin Preparation

The theabrownins were extracted and purified from Wuniuzao dark tea in accordance with the protocol established by Huang et al. [12]. The prepared tea extract was subsequently subjected to freeze-drying at −55 °C for a duration of 48 h, leading to the formation of theabrownins. And the obtained theabrownins were carefully stored at −20 °C for subsequent experiments.

### 2.2. Animal Experimental Design and Sample Collection

Male SPF-grade C57BL/6 mice, aged eight weeks, were acquired from Hangzhou Ziyuan Experimental Animal Technology Co., Ltd. (Hangzhou, China), under the license number SYXK (Zhejiang) 2018-0009. After a week of adaptive feeding, a total of 50 mice with similar weight were randomly assigned into five groups, ten mice per group: normal control group (NC), high-fat diet group (HFD), positive control group (PC), low-dose theabrownin group (TBL), and high-dose theabrownin group (TBH). The mice in NC group were fed a regular diet (Wuxi Fanbo Biotechnology Co., Ltd., Wuxi, China), while those in the groups of HFD, PC, TBL, and TBH were provided with a high-fat diet (D12492, Wuxi Fanbo Biotechnology Co., Ltd.) containing 60% of calories derived from fat. The mice were administered a daily oral gavage volume of 0.01 mL/g, with the positive control group administered atorvastatin calcium tablets at a dose of 10 mg/kg·BW. The TBL (150 mg/kg·BW) and TBH (300 mg/kg·BW) groups, as established per the data acquired by Huang et al. (2019) [12], were administered different doses of theabrownins. In contrast, the NC and HFD groups were orally gavaged with an equal dose of normal saline. During the experiment, food and water were available ad libitum to all mice. Weekly measurement of their weight was taken. Following a 9-week treatment period, the mice were subjected to a 12 h fasting period and euthanized using carbon dioxide inhalation. The liver was weighed and stored under appropriate conditions, while colonic fecal samples were acquired and preserved at −80 °C for subsequent analysis.

### 2.3. Detection of Serum Lipid Levels and Enzyme Activity

After a 2 h incubation of plasma in EP tubes, the supernatant was acquired by 10 min centrifugation at 3500 rpm and 4 °C. The measurement of serum triglycerides (TG), total cholesterol (TC), low-density lipoprotein cholesterol (LDL-C), high-density lipoprotein cholesterol (HDL-C), alanine aminotransferase (ALT), and aspartate aminotransferase (AST) levels were performed per the provided protocol of the kit (Nanjing Jiancheng Bioengineering Institute, Nanjing, China).

### 2.4. Liver Section Staining

During the dissection process, a consistent tissue section from the right lobe of the mouse liver was obtained. The obtained tissue section was fixed in 10% neutral buffered formalin for a day to ensure proper fixation. Following the completion of the fixation, the tissue underwent embedding procedures for further processing. The samples were processed by embedding them in paraffin for sectioning and in a frozen state for cryosectioning. Subsequently, the paraffin-embedded sections were stained with H&E, while the frozen sections were subjected to Oil Red O staining. Tissue pathology examination and analysis were conducted on each section to observe and analyze the tissue characteristics.

### 2.5. RNA Extraction and Quantitative PCR Analysis

The total RNA was extracted from the liver through the MiniBEST Universal RNA Extraction Kit, and its concentration was determined using a microspectrophotometer. Total RNA was converted into single-stranded cDNA utilizing the PrimeScript™ RT reagent kit with gDNA Eraser (Perfect Real Time). Real-time PCR II (Tli RNaseH Plus) utilized the TB Green^®^ Premix Ex Taq™ for gene expression analysis. The reagents for RNA extraction, reverse transcription, and q-PCR were procured from Takara Bio Inc., Beijing, China. The q-PCR reactions were conducted with these specific parameters: an initial step (denaturation) at 95 °C for 30 s, followed by 40 amplification cycles (95 °C for 5 s, 60 °C for 30 s, with fluorescence signal collection), and a melting curve analysis from 65 °C to 95 °C. The glyceraldehyde-3-phosphate dehydrogenase (GAPDH) was utilized as an internal reference for normalization. The relative expression levels of every gene were assessed through the comparative cycle threshold (ΔΔCt) technique. Table 1 provides a list of primers. 

### 2.6. Protein Extraction and Western Blotting

To extract total protein from the liver tissue samples, a RIPA buffer containing a protein inhibitor mixture was used. The protein concentration of the samples was subsequently evaluated through the BCA protein assay kit. The RIPA Buffer was acquired from Thermo Fisher Scientific (Waltham, MA, USA), while the BCA kit was supplied by the Beyotime Biotechnology Co., Ltd. (Shanghai, China). After separating the samples (60 μg) using SDS-PAGE, these were shifted onto a PVDF membrane. To block the membrane, THEABROWNINST with 5% bovine serum albumin (BSA) was utilized followed by overnight incubation with the primary antibody (FAS, Abcam (Cambridge, UK) ab128856, 1:3000; p-ACC1, CST (Danvers, MA, USA) 1818, 1:1000; ACC1, CST 3676, 1:1000; SREBP1, Abcam ab28481, 1:2000; SCD, CST 2794, 1:2000; PPARγ, Abcam ab272718, 1:1000; PPARα, Abcam ab126285, 1:1000; FXR, Abcam ab155124, 1:2000; CYP7A1, Thermo Fisher PA5-100892, 1:500; CYP27A1, Thermo Fisher PA5-27946, 1:2000; CYP8B1, Proteintech (Rosemont, IL, USA) 24889-1-AP, 1:1000; LXR, Abcam ab24362, 1:1000; GAPDH, Abcam ab181602, 1:10,000) at 4 °C. Following that, a room temperature incubation for 1 h with the secondary antibody was carried out. By utilizing Image J software (v 1.8.0), the band densities were analyzed to quantify the relative expression levels of the target protein. These levels were determined using the following formula: relative expression = (density value of target protein/density value of reference protein) × 10^n^.

### 2.7. High-Throughput Sequencing of Gut Microbiota

By utilizing high-throughput sequencing of 16S rDNA, the diversity, richness, and community structure of the mice gut microbiota were analyzed. Total RNA was extracted from colonic fecal samples of the mice with further amplification of the V3-V4 region using certain primers (F: ACTCCTACGGGAGGCAGCA, R: GGACTACHVGGGTWTCTAAT). The obtained paired-end sequence data from high-throughput sequencing were combined into a single sequence tag by merging the overlapping regions between the sequences. Subsequently, the sequence tags were clustered into Amplicon Sequence Variants (ASVs) using the UCLUST algorithm available in the QIIME software (v1.80) package [13]. Taxonomic classification of the ASVs was performed by assigning them labels based on a bacterial classification database [14]. Alpha diversity indices of the microbial community in the samples were evaluated using Mothur 1.30. Additionally, the QIIME software program was employed to analyze the β diversity of the microbial community, assessing the relative abundance of different taxa at various taxonomic levels across the samples. Furthermore, the species composition obtained from ASV annotation through high-throughput sequencing was compared to the KEGG PATHWAY database [15] to perform functional annotation and analysis of different pathways.

### 2.8. Statistical Analysis

The data obtained from this experiment were subjected to a one-way analysis of variance in SPSS 27.0 (SPSS Inc., Chicago, IL, USA) for Windows. The differences between means were tested by Tukey’s multiple range test. The level of significance was chosen at *p* < 0.05. Plotting was performed with Origin 2022b software (OriginLab Corporation, Northampton, MA, USA). Values were presented as means with standard deviation.

## 3. Results

### 3.1. Theabrownin Reduced HFD-Induced Weight Gain and Hyperlipidemia

**Figure 1:**
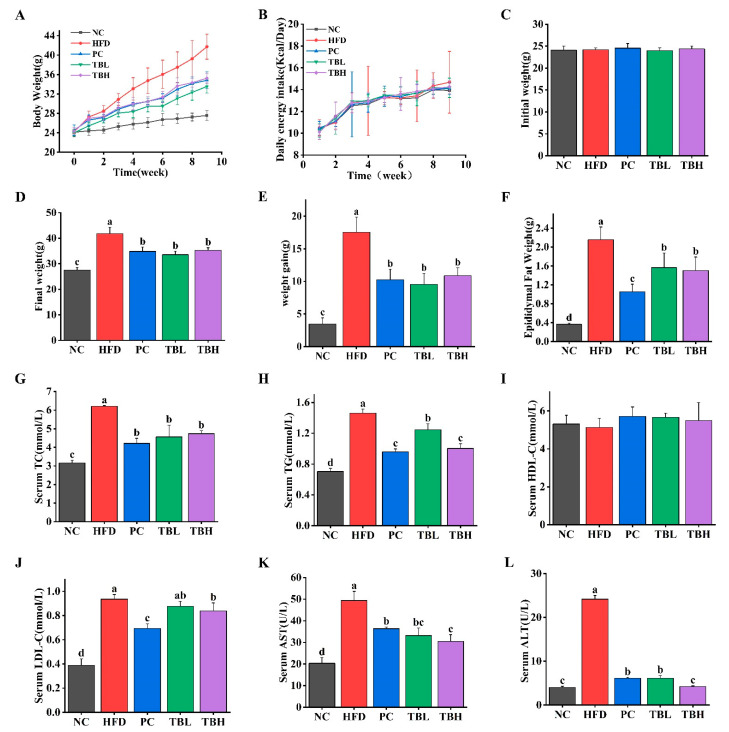
The effects of theabrownin on body weight, serum lipids, and activity of the two enzymes in mice fed high-fat diet. (**A**) Change trend of body weight. (**B**) Daily energy intake. (**C**) Initial body weight. (**D**) Final body weight. (**E**) Weight gain. (**F**) Epididymis fat weight. (**G**) Serum TC. (**H**) Serum TG. (**I**) Serum HDL-C. (**J**) Serum LDL-C. (**K**) Serum AST. (**L**) Serum ALT. Values are means ± SD, *n* = 8. ^a–d^ Distinct letters denote a significant variance in mean values (Tukey test, *p* < 0.05).

Figure 1A–F show the body weight change trend over time, the initial body weight, final body weight, body weight gain, and epididymal adipose weight of mice in five groups. There was no significant difference (*p* > 0.05) in the initial body weight of mice among the five groups. The weight of mice in the HFD group continued to increase over time, while the weight of mice in the PC, TBL and TBH group increased over time but was inhibited compared with that in the HFD group. The final body weight of mice in the HFD, PC, TBL and TBH group was significantly higher (*p* < 0.05) than that in the NC group. The final body weight of mice in the PC group and the two theabrownin groups was significantly lower (*p* < 0.05) than that in the HFD group. The body weight gain of mice in the HFD, PC, TBL and TBH group was significantly elevated (*p* < 0.05) than that in the NC group. The body weight gain of mice in the PC group and the two theabrownin groups was significantly inferior (*p* < 0.05) to that in the HFD group. In addition, the epididymal adipose weight of mice in the HFD, PC, TBL and TBH group was significantly higher (*p* < 0.05) than that in the NC group. The epididymal adipose weight of mice in the PC group and the two theabrownin groups was significantly lower (*p* < 0.05) than that in the HFD group. There was no significant difference in the daily energy intake of all groups of mice (*p* > 0.05), so the effect of energy intake on this study can be excluded.

The effects of theabrownin on serum TC, TG, HDL-C, LDL-C in mice fed a HFD are shown in Figure 1G–J. The serum TC level of mice fed HFD was significantly increased (*p* < 0.05) compared with the mice in NC group. The serum TC level of mice was significantly decreased (*p* < 0.05) in PC, TBL and TBH group compared with the HFD group. Moreover, the serum TG level of mice fed HFD was significantly higher (*p* < 0.05) than that of the NC group. The serum TG level of mice in PC, TBL and TBH group was significantly lower (*p* < 0.05) than that in HFD group. There was no significant difference (*p* > 0.05) in the serum HDL-C level of mice among five groups. The serum LDL-C level of mice fed HFD was significantly increased (*p* < 0.05) compared with the mice in NC group. The serum LDL-C level of mice in the PC and TBH groups was significantly decreased (*p* < 0.05) compared with the mice in NC group. 

Figure 1K,L shows the activity of AST and ALT in the mice in the five groups. The activity of AST in mice fed a HFD was significantly increased (*p* < 0.05) compared with mice in the NC group. The activity of AST in the PC, TBL and TBH group was significantly decreased (*p* < 0.05) compared with that in the HFD group. In addition, the activity of ALT in the PC, TBL and TBH groups was significantly decreased (*p* < 0.05) compared with that in the HFD group. The activity of ALT in the PC and TBL group was significantly higher (*p* < 0.05) than that in the NC group, whereas there was no significance (*p* > 0.05) in the activity of ALT between TBH group and NC group.

### 3.2. Theabrownin Reduced HFD-Induced Lipid Deposition in Liver

**Figure 2:**
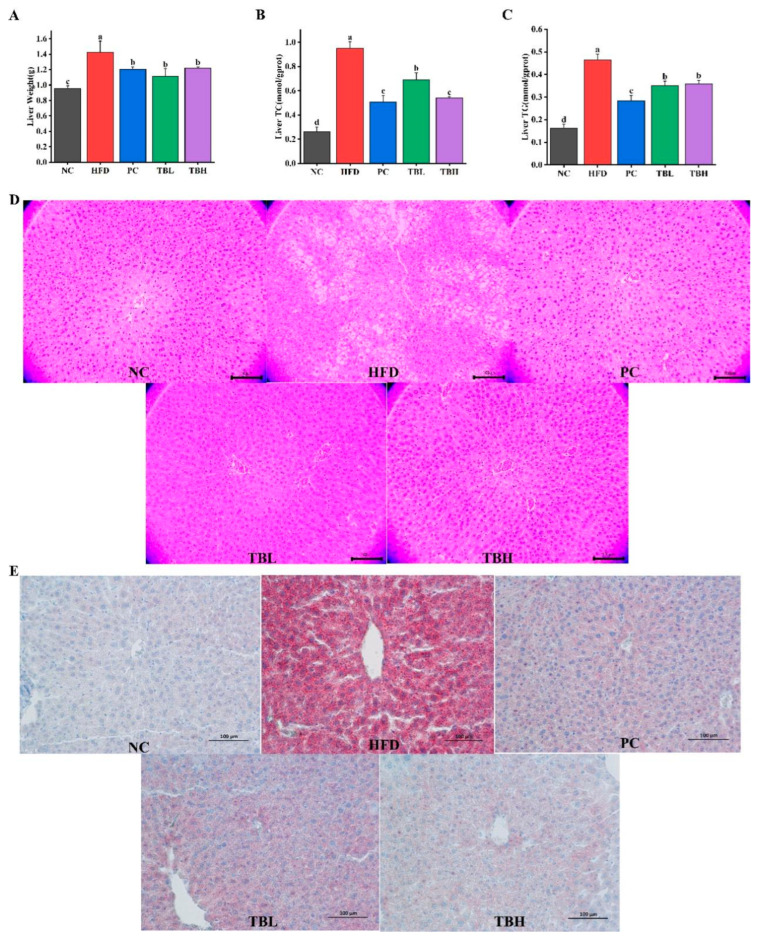
The effects of theabrownin on hepatic lipid deposition induced by high-fat diet in mice. (**A**) Liver weight. (**B**) Hepatic TC. (**C**) Hepatic TG. (**D**) Representative HE staining of livers from five groups (200×, scale bar = 100 μm). (**E**) Representative Oil-Red-O staining of liver sections from five groups (200×, scale bar = 100 μm). Values are means ± SD, *n* = 8. ^a–d^ Distinct letters denote a significant variance in mean values (Tukey test, *p* < 0.05).

Figure 2A–C display the liver weight, levels of hepatic TC and TG of mice in five groups. The liver weight, levels of hepatic TC and TG of mice fed HFD was significantly elevated (*p* < 0.05) compared with the mice fed a normal diet. Nevertheless, the liver weight, levels of hepatic TC and TG of mice in the PC, TBL, and TBH group was significantly decreased (*p* < 0.05) compared with that in the HFD group.

Figure 2D,E exhibit the effects of theabrownin on liver histology in mice fed HFD. The condition of liver was evaluated by H&E staining (Figure 2D) and Oil-Red O staining (Figure 2E). In the NC group, the hepatocytes were arranged in radial order along the central vein, the cell outline was clear, and the lipid droplets were few and small. However, the hepatocytes in the HFD group were disorganized, with unclear boundaries and significantly increased lipid droplets. Additionally, nuclear condensation was observed, indicating typical characteristics of fatty deposits along with liver damage. In addition, the histological morphology of the liver nearly returned to their normal state in the PC group and two theabrownin groups. And the effect of high dose theabrownin is better than that of the drug and low-dose theabrownin visibly.

### 3.3. Theabrownin Regulated Relative Expression of Lipid Metabolism-Related Genes in Mice

**Figure 3:**
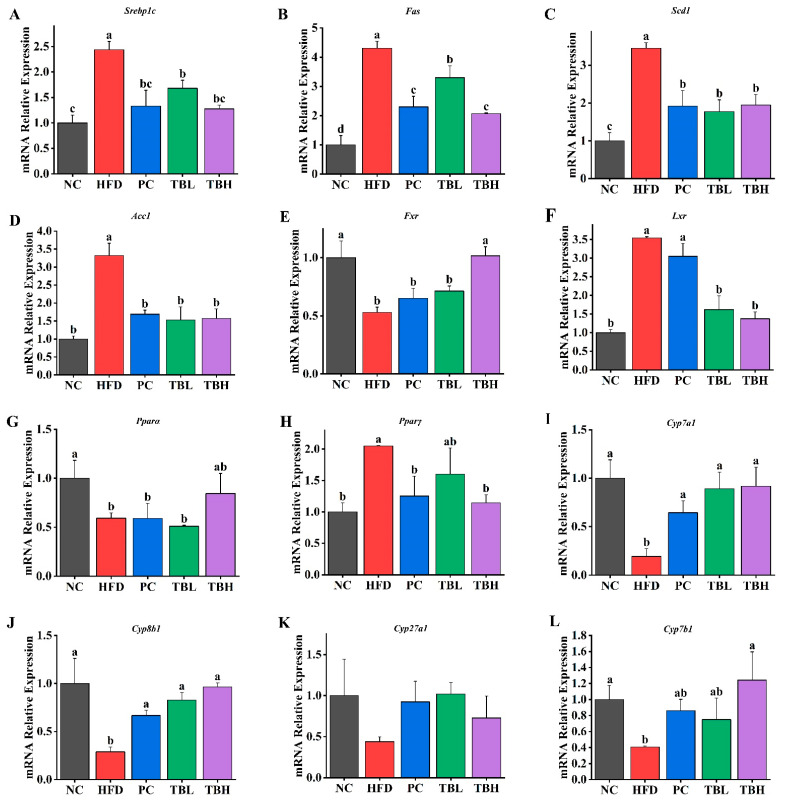
The mRNA relative expression of genes related with lipid metabolism in liver of mice in five groups. (**A**) *Srepb1c*; (**B**) *Fas*; (**C**) *Scd1*; (**D**) *Acc1*; (**E**) *Fxr*; (**F**) *Lxr*; (**G**) *Pparα*; (**H**) *Pparγ*; (**I**) *Cyp7a1*; (**J**) *Cyp8b1*; (**K**) *Cyp27a*; (**L**) *Cyp7b1*. ^a–d^ Means sharing no common superscripts differ significantly (Tukey test, *p* < 0.05).

Figure 3 shows the mRNA relative expression of genes related to lipid metabolism in the liver of mice in five groups. It was observed, as shown in Figure 3A–D, that the gene expression of *Srepb1c*, *Fas*, *Scd1* and *Acc1* was significantly upregulated (*p* < 0.05) in the HFD group compared with that in the NC group. The gene expression of *Srepb1c*, *Fas*, *Scd1* and *Acc1* was significantly downregulated (*p* < 0.05) in the PC, TBL, and TBH group compared with that in the HFD group. 

Figure 3E displays that the gene expression of *Fxr* was significantly downregulated (*p* < 0.05) in the HFD group compared with that in the NC group. There was no significance (*p* > 0.05) in the gene expression of *Fxr* among the HFD, PC and TBL group, whereas the gene expression of *Fxr* was significantly upregulated (*p* < 0.05) in the TBH group. Figure 3F shows that the gene expression of *Lxr* was significantly downregulated (*p* < 0.05) in the HFD group compared with that in the NC group. And the gene expression of *Lxr* was significantly upregulated (*p* < 0.05) in the PC, TBL, and TBH group compared with that in HFD group, whereas there was no significance (*p* > 0.05) in the gene expression of *Lxr* among the NC, PC, TBL and TBH groups. The *Pparα* gene expression was significantly downregulated (*p* < 0.05) in the HFD, PC, and TBL group compared with the NC group, whereas there was no significance (*p* > 0.05) between the TBH and NC group (Figure 3G). The gene expression level of *Pparγ* in the HFD group was significantly higher (*p* < 0.05) than the NC group, whereas there was no significance (*p* > 0.05) among the NC, PC, and TBH groups (Figure 3H). 

The gene expression of *Cyp7a1*, *Cyp8b1* and *Cyp7b1* was significantly downregulated (*p* < 0.05) in the HFD group compared with the NC group, whereas there was no significance (*p* > 0.05) among the NC, PC, TBL and TBH groups (Figure 3I,J,L). Nevertheless, there was no significance (*p* > 0.05) in gene expression of *Cyp8b1* among the five groups.

### 3.4. Theabrownin-Regulated Relative Expression of Lipid Metabolism-Related Key Proteins in Mice

**Figure 4:**
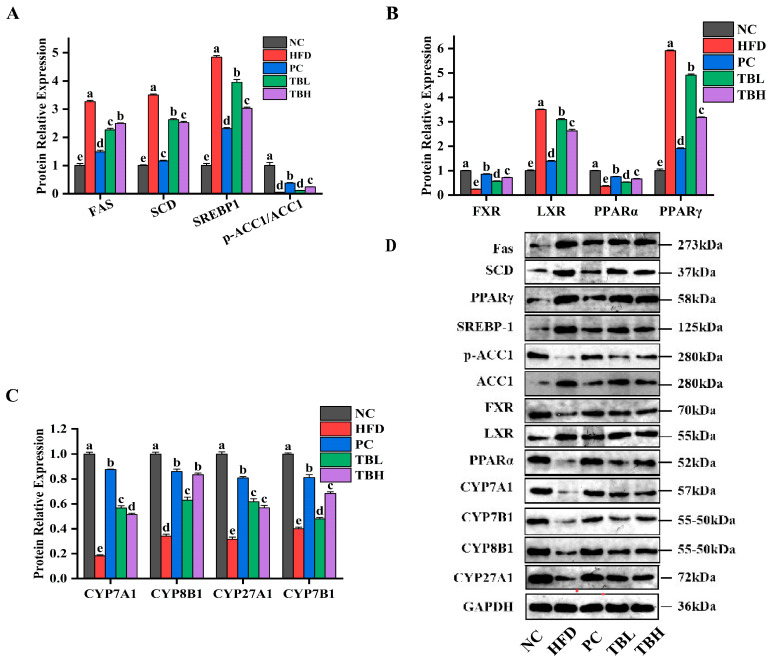
The expression levels of proteins associated with liver lipid metabolism pathways in each group of mice. (**A**) Downstream proteins of SREBP; (**B**) upstream proteins of SREBP; (**C**) proteins related to bile acid metabolism; (**D**) protein electrophoresis image. ^a–e^ Distinct letters denote a significant variance in mean values (Tukey test, *p* < 0.05).

Figure 4 shows the relative expression of proteins related with lipid metabolism in the liver of mice in five groups. It was observed in Figure 4A that the protein expression levels of FAS, SCD and SREBP1 were significantly higher (*p* < 0.05) in mice fed a HFD than those in the NC group, while the protein expression levels of FAS, SCD and SREBP1 were significantly lower (*p* < 0.05) in the HFD group than those in the PC, TBL and TBH groups. On the contrary, the protein expression level of phosphorylated ACC1 was significantly lower (*p* < 0.05) in mice fed a HFD than those in the NC group. And the protein expression level of phosphorylated ACC1 was significantly higher (*p* < 0.05) in the PC, TBL and TBH groups than those in the HFD group.

Figure 4B shows that the protein expression levels of FXR and PPARα were significantly lower (*p* < 0.05) in mice fed HFD than those in NC group. And the protein expression levels of FXR and PPARα were significantly higher (*p* < 0.05) in the PC, TBL and TBH groups than the HFD group. In addition, the protein expression levels of LXR and PPARγ were significantly higher (*p* < 0.05) in mice fed a HFD than those in the NC group. And the protein expression levels of LXR and PPARγ were significantly lower (*p* < 0.05) in the PC, TBL and TBH groups than the HFD group. 

Figure 4C shows that the protein expression levels of CYP7A1, CYP8B1, CYP27A1 and CYP7B1 were significantly lower (*p* < 0.05) in mice fed a HFD than those in the NC group. However, the protein expression levels of CYP7A1, CYP8B1, CYP27A1 and CYP7B1 were significantly higher (*p* < 0.05) in the PC, TBL and TBH groups than the HFD group.

### 3.5. Theabrownin-Adjusted Gut Microbiota Composition in Mice

**Figure 5:**
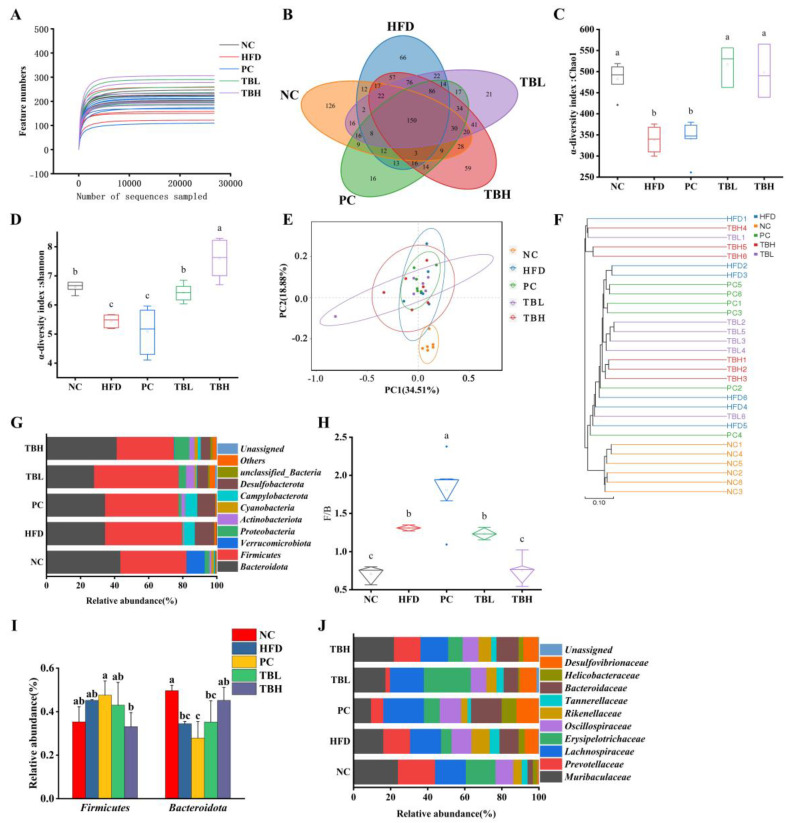
The effects of theabrownin on gut microbiota in mice fed a high-fat diet. (**A**) Sequencing depth; (**B**) Venn diagram; (**C**) Chao1 index; (**D**) Shannon index; (**E**) PCA analysis; (**F**) UPGMA clustering analysis; (**G**) relative abundance of bacterial groups at the phylum level; (**H**) ratio of Firmicutes to Bacteroidetes; (**I**) relative abundance of Firmicutes and Bacteroidetes; and (**J**) relative abundance of bacterial groups at the family level. ^a–c^ Distinct letters denote a significant variance in mean values (Tukey test, *p* < 0.05).

As shown in Figure 5, 16S rDNA sequencing analysis in the V3-V4 region was performed on 30 colon stool samples from five groups (each group of six qualified samples). The rarefaction curves (Figure 5A) gradually rose and then entered a plateau, indicating that there was sufficient OTU coverage to accurately describe the microbiological composition of each group. In the Venn diagram (Figure 5B), overlapping parts represent the number of OTUs shared by groups, and non-overlapping parts are the number of OTUs unique to the groups. The Venn diagram indicates that the five groups had 150 common OTUs, and the NC group had the most unique OTUs. 

Compared to the NC group, the colonic microbiota in mice in the HFD and PC group had reduced richness (*p* < 0.05) based on the Chao1 index, and had less diversity (*p* < 0.05) based on the Shannon index (Figure 5C,D). However, the colonic microbiota in mice in the TBL and TBH groups had greater richness (*p* < 0.05) than that in the NC group, whereas there was no significance (*p* > 0.05) in microbial richness among the NC, TBL and TBH groups (Figure 5C). Moreover, there was no significance (*p* > 0.05) in the colonic microbiota between the NC and TBL groups, and the colonic microbiota in TBH had more diversity (*p* < 0.05) based on the Shannon index (Figure 5D).

The β-diversity analyses were performed using principal component analysis (PCA), with PC1 accounting for 34.51% and PC2 for 18.88% of the variation and clustering analysis (Figure 5E,F), which revealed that the NC group and the HFD group were obviously separated. After low and high doses of theabrownin intervention, the colonic microbiota gradually became closer to that of the NC group.

The relative abundance of colonic microbiota at the levels of the phylum and family was further analyzed (Figure 5G,I,J). Firmicutes and Bacteroidota were the dominant phyla of colonic microbiota in mice. There was no significance (*p* > 0.05) in the abundance of Firmicutes among the NC, TBL and TBH groups. The abundance of Bacteroidota was significantly increased in the HFD group compared with the NC group, while there was no significance (*p* > 0.05) in the abundance of Bacteroidota between the NC and TBH groups. Compared to the NC group, the ratio of Firmicutes and Bacteroidota (F/B) was significantly increased (*p* < 0.05) in the HFD group. Nevertheless, there was no significance (*p* > 0.05) in F/B between the NC and TBH groups.

**Figure 6:**
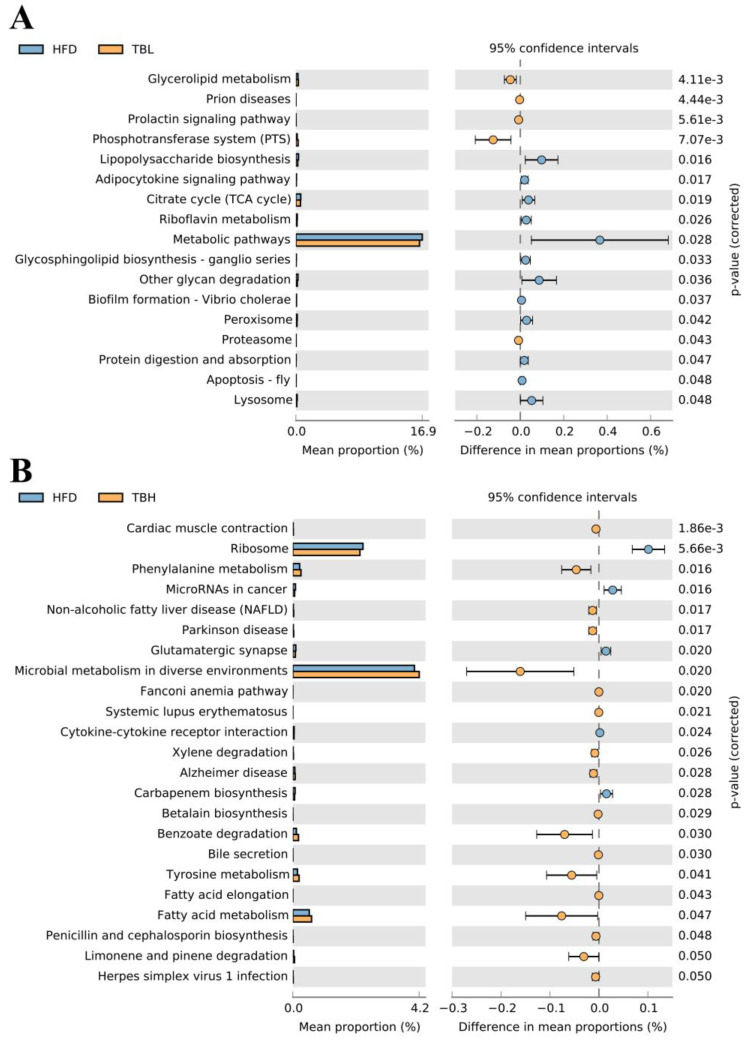
The differences in functional genes of microbial communities in metabolic pathways among different groups by analyzing the composition and differences of KEGG metabolic pathways. (**A**) Analysis of KEGG metabolic pathway differences between the TBL and HFD group. (**B**) Analysis of KEGG metabolic pathway differences between the TBH and HFD group.

A total of 17 significantly altered (*p* < 0.05) KEGG metabolic pathways were identified between the TBL and HFD groups (Figure 6A). Among these pathways, five pathways such as glycerolipid metabolism exhibited increased functional abundance in TBL group, whereas 12 pathways (e.g., lipopolysaccharide biosynthesis) showed decreased abundance. These changes were observed in energy metabolism-related pathways, including triglyceride metabolism, adipocytokine signaling pathway, peroxisome, and protein digestion and absorption. A total of 22 significantly altered (*p* < 0.05) KEGG metabolic pathways were identified between the TBH and HFD group (Figure 6B). Therein, 17 pathways, such as fatty acid metabolism, exhibited a significant increase in functional abundance in the TBH group, whereas five pathways (e.g., microRNA in cancer) showed a significant decrease. These changes encompassed various aspects of lipid metabolism and bile acid synthesis, including NAFLD, bile secretion, fatty acid elongation and metabolism.

## 4. Discussion

The prevalence of obesity has emerged as a worldwide health issue, and liver steatosis linked with obesity can worsen the progression of obesity and even lead to liver cancer [16]. Therefore, investigating the mechanism of theabrownin from Wuniuzao dark tea in alleviating liver steatosis, preventing obesity, and regulating gut microbiota is of significant importance. Previous studies on animals have confirmed that mice with obesity induced by high-fat diet typically exhibit symptoms of hyperlipidemia, significant weight gain, and liver enlargement accompanied by steatosis and damage. The results from the current study also prove the point. Elevated blood lipid levels are typical symptoms of obesity-related metabolic disorders stimulated by a high-fat diet [17]. LDL-C transports synthesized cholesterol to various tissues in the body, while HDL-C redistributes cholesterol from peripheral tissues to the liver for breakdown and metabolism. The levels of both serum LDL-C and HDL-C have been found to be associated with metabolic disorders related to obesity [18]. In our present study, the levels of serum TG, TC and LDL-C were significantly increased in mice fed a HFD, suggesting that these mice exhibited symptoms of metabolic disorders related to obesity. In addition, the reduced levels of TG, TC and LDL-C in the two theabrownin groups indicates that theabrownin could relieve the symptoms of obesity, which aligns with a previous study on theabrownin from Pu-erh tea [12]. The enzyme activity of serum AST and ALT is indicative of the normal function of the liver [19]. In the current study, the results showed that the high-fat diet caused liver damage, while the activity of serum AST and ALT was lower in two theabrownin groups than that in HFD group, which suggests theabrownin could improve liver function to some extent. In addition, the results of the experiment also suggest that theabrownin has similar effects to atorvastatin, a weight-reducing drug, in improving serum lipid levels, the enzyme activity of AST and ALT, and liver function damage in obese mice. In other words, theabrownin from Wuniuzao dark tea could safely and effectively replace diet drugs and play a role in alleviating obesity.

The liver is a crucial site for lipid metabolism, playing a role in regulating the process of lipid metabolism and promoting the conversion of fat in the body. Lipid accumulation is primarily influenced by the synthesis, transport, and oxidation of fatty acids, as well as glycolysis and gluconeogenesis. *Srebps* have the ability to regulate the production and transport of fatty acids and cholesterol [20]. Belonging to the *Srebp* transcription factor family [21], *Srebp1c* is highly expressed in liver. It is strongly involved in the activation of *Acc, Fas, and Scd*, thereby influencing de novo fatty acid synthesis in the liver. The impact of tea on the *Srebp* pathway involved in lipid metabolism has been extensively studied, and compelling evidence suggests that tea exerts significant effects. The active constituents found in tea have been shown to modulate the expression of *Srebp* and its upstream genes (*Ampk*, *Pparγ*, *Fxr*, and *Lxr*), as well as its downstream genes (*Acc*, *Fas* and *Scd*). These regulatory effects result in the inhibition of fatty acid synthesis, reduction in fatty acid unsaturation, and attenuation of lipid accumulation [22,23]. In the present study, the expression levels of *Srepb1c* both in transcription and translation were significantly upregulated in the HFD group compared with that in the NC group, while the expression levels were significantly downregulated in the PC, TBL, and TBH groups compared with that in the HFD group, which was consistent with previous studies in which tea extracts from Pu-erh and Hawk demonstrated significant effects on the expression of *Srebp1c*, resulting in decreased levels of TC and enhanced lipid oxidation in the liver [24,25]. ACC, a primary rate-limiting enzyme involved in the synthesis of fatty acid, performs a crucial function in limiting fatty acid entry into mitochondria for oxidation. Both FAS and SCD, as important factors in de novo lipogenesis, have been proven to be associated with the development of obesity. In the current study, the expression levels of *Fas* and *Scd* both in transcription and translation were significantly upregulated in the HFD group compared to the NC group, while their expression levels were significantly downregulated in the PC, TBL, and TBH groups comparing to the HFD group, indicating that the HFD promoted fatty acid synthesis, while theabrownin as well as diet drug could inhibit fatty acid synthesis.

The expression level of *Lxr*, an upstream regulator of *Srebp1c* in hepatic lipid metabolism, correlates with the severity of NAFLD symptoms. The suppression of *Lxr* expression leads to the decreased expression of downstream targets (e.g., *Srebp-1c*, *Fas*, *Scd-1*, and *Acc1*) and reduces de novo lipogenesis in the liver [26]. Inversely, the activation of *Fxr* expression can effectively suppress hepatic lipid synthesis by activating the *Fxr-Shp-Srebp-1c* pathway. Moreover, it can also enhance the expression of *Pparα* and *Cpt1*, leading to increased fatty acid oxidation and reduced hepatic fatty acid uptake [27]. Perturbation in *Pparα* expression, a crucial regulator of hepatic metabolism, is involved in the pathogenesis of various liver conditions such as hepatic steatosis, steatohepatitis, fibrosis, and hepatocarcinogenesis [28]. The results observed from the current research revealed that the de novo lipogenesis in liver was reduced due to theabrownin as well as the diet drug, and the TBH had the better effects overall.

As the major byproducts of cholesterol metabolism, bile acids perform a crucial function in maintaining the overall balance of cholesterol in body through their synthesis and elimination processes. The classic pathway of hepatic bile acid metabolism involves the pivotal role of CYP7A1, which acts as the rate-limiting enzyme for cholesterol breakdown and catalyzes bile acid synthesis. Together with CYP8B1, it forms a crucial enzymatic system in liver [29,30]. Furthermore, an alternative pathway for bile acid metabolism is constituted by intramitochondrial enzymes, namely CYP27A1 and CYP7B1. However, it is important to note that this pathway contributes to less than 6% of the total bile acid synthesis in the human body. The present study exhibited a significant impact of theabrownin on the transcription and translation levels of bile acid metabolism-related genes in mice. The results observed suggests that theabrownin could reduce hepatic cholesterol accumulation by facilitating hepatic bile acid metabolism. Moreover, it was noted that the regulatory effects of theabrownin on bile acid metabolism primarily occurred through the classical pathway, which were congruent with prior research findings [12]. Based on the results of this study, it can be concluded that theabrownin had the ability to modulate the hepatic bile acids metabolism, leading to enhanced cholesterol degradation and alleviation of hepatic steatosis in mice with obesity.

In the realm of obesity research, the regulatory role of gut microbiota has garnered great attention. Within the human gut microbiota, the prevailing bacterial taxa comprise Bacteroidetes, Firmicutes, Actinobacteria, and Proteobacteria. Notably, Bacteroidetes and Firmicutes emerge as the predominant phyla, collectively accounting for approximately 90% of the gut bacterial population [31]. The gut environment maintains a dynamic equilibrium between different microbial communities and their interaction with the host. Among these communities, Bacteroideta and Firmicutes, which are considered as obesity-associated gut microbiota, play a significant role in the development of obesity [32]. In host metabolism, Firmicutes play a significant role by breaking down dietary fiber and generating butyrate, a compound that has important implications. Butyrate enhances insulin sensitivity, regulates energy metabolism, and possesses anti-inflammatory and immune-modulatory properties [33]. In addition, it is worth noting that butyrate acts as an activator of *Pparγ* [34]. The relative abundance of Firmicutes and Bacteroidetes, two prominent bacterial phyla, is intricately linked to weight gain, gut inflammation, and the progression of obesity. The F/B serves as a biomarker for disrupted gut microbiota in obesity, contributing to imbalanced energy metabolism and an increased incidence of obesity-related metabolic syndrome [35,36]. The results noted in our study revealed that the F/B ratio in the HFD group was markedly enhanced, suggesting that disrupted energy metabolism and increased incidence of hepatic steatosis would occur in the HFD group. Conversely, the mice in the TBH group depicted a notable reduction in the F/B comparing to HFD group, resembling that in the NC group, which indicates that TBH could reduce incidence of obesity-related metabolic syndrome. Furthermore, through functional prediction analysis of KEGG metabolic pathways, it was also observed that theabrownin exerted effects on the pathways associated with lipid metabolism and bile acid metabolism. However, although we have preliminarily revealed the regulatory effect of theabrownin on the gut microbiota of obese mice, the specific bacterial genus or metabolite through which the gut microbiota regulates the lipid metabolism pathway needs further in-depth research. 

## 5. Conclusions

In conclusion, the theabrownin from Wuniuzao dark tea could regulate the SREBP lipid metabolism pathway and bile acid metabolism process, and affect the gut microbiota composition of mice, in order to alleviate liver steatosis and liver injury in mice fed a high-fat diet, achieving the liver protection and anti-obesity effects.

## Figures and Tables

**Table 1:** Primers and conditions used in real-time polymerase chain reaction.

Gene	Primer Sequences (5′ to 3′)	Size (bp)	Accession Number
*Gapdh*	GAAGGTCGGTGTGAACGGATTTG	127	NM_001289726.2
	CATGTAGACCATGTAGTTGAGGTCA		
*Scd1*	GCAAGCTCTACACCTGCCTCTTC	110	NM_009127.4
	CAGCCGTGCCTTGTAAGTTCTG		
*Cd36*	GGAGCCATCTTTGAGCCTTCA	217	NM_001159556.2
	GAACCAAACTGAGGAATGGATCT		
*Acc1*	CGTGCAATCCGATTTGTTGTCATG	86	NM_133360.3
	GGAACATAGTGGTCTGCCATCT		
*Srebp1c*	CCCGGCTATTCCGTGAACAT	111	XM_006532716.4
	GCAGATATCCAAGGGCATCTGA		
*Fas*	GCTTTGCTGCCGTGTCCTTCTA	102	NM_007988.3
	CTGTCTTGGCACGCAGCAGT		
*Fxr*	CTGGCATCTATGAACTCAGGC	96	NM_001385711.1
	CCATTCGCGGCTTCTTTGT		
*Lxr*	ACAGAGCTTCGTCCACAAAAG	165	XM_006499168.4
	GCGTGCTCCCTTGATGACA		
*Pparα*	AACATCGAGTGTCGCGAATATGTGG	99	XM_030248424.2
	CCGAATAGTTCGCCGAAAGAA		
*Pparγ*	CTCCAAGAATACCAAAGTGCGA	150	NM_011146.4
	GCCTGATGCTTTATCCCCACA		
*Cyp7a1*	GGGATTGCTGTGGTAGTGAGC	100	NM_007824.3
	GGTATGGAATCAACCCGTTGTC		
*Cyp7b1*	GGAGCCACGACCCTAGATG	164	NM_007825.5
	GCCATGCCAAGATAAGGAAGC		
*Cyp8b1*	CTAGGGCCTAAAGGTTCGAGT	111	NM_010012.3
	GTAGCCGAATAAGCTCAGGAAG		
*Cyp27a1*	CCAGGCACAGGAGAGTACG	139	NM_024264.5
	GGGCAAGTGCAGCACATAG		

*Gapdh*, glyceraldehyde-3-phosphate dehydrogenase; *Scd1*, stearoyl-CoA desaturase1; *Cd36*, cluster of differentiation 36; *Acc1*, acetyl-CoA carboxylase1; *Srebp*, sterol regulatory element binding protein; *Fas*, fatty acid synthase; *Fxr*, farnesoid X receptor; *Lxr*, liver X receptor; *Ppar*, peroxisome proliferator-activated receptor; *Cyp7a1*, cholesterol 7-alpha hydroxylase; *Cyp7b1*, cholesterol 7-beta hydroxylase; *Cyp8b1*, cholesterol 8-beta hydroxylase; *Cyp27a1*, cholesterol 27-alpha hydroxylase.

## Data Availability

Data are contained within the article.

## Abbreviations

*Acc1*, acetyl-CoA carboxylase1; ALT, alanine aminotransferase; AST, aspartate aminotransferase; ASVS, amplicon sequence variants; *Cyp7a1*, cholesterol 7-alpha hydroxylase; *Cyp7b1*, cholesterol 7-beta hydroxylase; *Cyp8b1*, cholesterol 8-beta hydroxylase; *Cyp27a1*, cholesterol 27-alpha hydroxylase; *Fas*, fatty acid synthase; F/B, Firmicutes to Bateroidota; *Fxr*, farnesoid X receptor; *Gapdh*, glyceraldehyde-3-phosphate dehydrogenase; H&E, hematoxylin and eosin; HDL-C, high-density lipoprotein cholesterol; HFD, high-fat diet; LDL-C, low-density lipoprotein cholesterol; *Lxr*, liver X receptor; NC, normal chow diet; PC, positive drug-control group; *Ppar*, peroxisome proliferator-activated receptor; *Srebp*, sterol regulatory element binding protein; *Scd1*, stearoyl-CoA desaturase1; TC, total cholesterol; TG, triglyceride; TBH, high-dose theabrownin; TBL, low-dose theabrownin.
